# An integrated study for seismic structural interpretation and reservoir estimation of Sawan gas field, Lower Indus Basin, Pakistan

**DOI:** 10.1016/j.heliyon.2023.e15621

**Published:** 2023-04-20

**Authors:** Muhsan Ehsan, Muhammad Arslan Shakeel Toor, Muhammad Iqbal Hajana, Nadhir Al-Ansari, Amjad Ali, Ahmed Elbeltagi

**Affiliations:** a Department of Earth and Environmental Sciences, Bahria School of Engineering and Applied Sciences, Bahria University, Islamabad, 44000, Pakistan; b Civil, Environmental and Natural Resources Engineering, Lulea University of Technology, 97187, Lulea, Sweden; c School of Earth Sciences, Zhejiang University, Hangzhou, Zhejiang, China; d Agricultural Engineering Dept., Faculty of Agriculture, Mansoura University, Mansoura 35516, Egypt

**Keywords:** Lower Goru formation, Seismic data interpretation, Seismic attributes, Petrophysical analysis, Rock physics, Contour mapping, Hydrocarbon bearing zones

## Abstract

The information about the subsurface structure, type of fluids present in the reservoir, and physical properties of the rocks is essential for identifying potential leads. The integrated approach of petrophysical analysis, seismic data interpretation, seismic attributes analysis, lithology, mineralogy identification, and Gassmann fluid substitution were used for this purpose. The structural interpretation with the help of seismic data indicated the extensional regime with horst and graben structures in the study area. The two negative flower structures are cutting the entire Cretaceous deposits. The depth contour map also indicate favorable structures for hydrocarbon accumulation. The four possible reservoir zones in the Sawan-01 well and two zones in the Judge-01 well at B sand and C sand levels are identified based on well data interpretation. The main lithology of the Lower Goru Formation is sandstone with thin beds of shale. The clay types confirm the marine depositional environment for Lower Goru Formation. The water substitution in the reservoir at B sand and C sand levels indicated increased P-wave velocity and density. The water substitution affected the shear wave velocity varies slightly due to density changes. The cross plots of P-impedance versus Vp/Vs ratio differentiate the sandstone with low P-impedance and low Vp/Vs ratio from shaly sandstone with high values in the reservoir area. The P-impedance and S-impedance cross plot indicate increasing gas saturation with a decrease in impedance values. The low values of Lambda-Rho and Mu-Rho indicated the gas sandstone in the cross plot.

## 1 Introduction

Previous studies related to Lower Goru Formation in Lower Indus Basin (LIB), Pakistan, have indicated that the spatial distribution of petrophysical properties is still problematic due to its heterogeneous nature [1]. The primary purpose of geophysical studies is to imaging the subsurface to recognize the prospect. These studies are used to gather rock and fluid properties inside those identified prospects. Significantly, no single geophysical method can give a complete image of the subsurface and its properties. Therefore, the newly combined interpretation of multiple data types has become a much-considered topic. Specifically, the integration of seismic, rock physics and well log data analysis can enhance assurance with which the reservoir lithology and fluid properties are derived [2]. The integration of different techniques is not a new idea. Therefore, this integration of geophysical methods is used in this study for reservoir characterization to improve the analysis.

In 1989 a gas discovery in sands of the Lower Goru Formation (LGF) at Kadanwari opened new play in the LIB. Several gas discoveries, including Miano, Mari Deep and Rehmat, were made from the formation mentioned earlier. Sawan Gas Field of OMV is also producing from the sands of the LGF (OMV, Eni.). Miano, Latif, Gambat, and Tajjal of PPL are the surrounding fields to the Sawan Gas Field. After drilling of Sawan-1 and Sawan-2 wells in 1997, Sawan Gas Field was opened for production. There are 16 wells drilled in this field, but only 14 wells are producing here. The field is commercially producible with 1534 BCF gas reserves. This field is a joint venture of OMV, Pakistan Petroleum Limited (PPL) and ENI Pakistan with an average daily production of 61.7 MMscfd gas [3]. After initial discovery, a 1000-line kilometer of 2D seismic data and 300 sq. kilometer of 3D seismic data was reprocessed. This field entered an intense phase in 2002 after the drilling of the Sawan-4 well. OMV is the largest international gas-producing company in Pakistan after discovering this field [4].

The study area is the part of the great Indus Basin and is located at its southern part [5]. The sand intervals of LGF are widely spread in the LIB. These sand facies are proven reservoirs in the study area. The LGF sands units are deep in-depth and extremely heterogeneous due to interbedded shale units, making it difficult to estimate the reservoir properties. Petrophysical analysis and rock physics analysis were performed to understand the reservoir properties of the LGF. Seismic structural interpretation will be used to delineate the subsurface features.

The petrophysical analysis deals with the properties of porous media such as porosity, permeability, water saturation, fluid identification, resistivity, shaliness particularly in reservoir rock and contained fluids. These properties and their relationships are generally used to identify and assess reservoir rock, source rock and caprock. It is done mainly to estimate recoverable hydrocarbons. It is also used to relate seismic properties to the reservoir. The primary benefits of petrophysical analysis are improved well-to-seismic ties and more reliable models of seismic response due to reservoir changes (vertically laterally, and temporally). This improved interpretation can reduce drilling risk, enhance field productivity, and ultimately increase asset value [[6], [7], [8]].

Seismic attribute analysis is used to improve the spatial prediction of structural features from seismic data. Rock physics models are utilized to provides the linkage between reservoir properties and petrophysical parameters [[9], [10], [11]]. Rock physics analysis helps us understand the behavior of the reservoir and non-reservoir zones and correct some of the problems encountered in well log data. Fluid substitution is a significant segment of rock physics interpretation and the Gassmann equation supports it. That is based on the relationship of dry rock and saturated rock velocities [12,13]. Clay minerals play a significant role in reservoir performance and production. The presence of clay minerals in subsurface reservoirs reduces the effective porosity and permeability. To identify lithology and clay mineralogy more precisely of a formation, a combination of other log data and natural gamma ray logs [14,15].

The research aims to estimate the hydrocarbon potential with the help of seismic structure interpretation, seismic attributes analysis, lithology, and mineralogy identification, petrophysical analysis, rock physics analysis and fluid substitution. The research will lead to a better understanding of reservoir properties and enriched estimation of hydrocarbons existing in the area. The petrophysical analysis will provide fluid information present in the rocks. The rock physics analysis allows for predicting seismic properties away from the well. Rock physics study is carried out for analyzing the influence of porosity and saturation variations on the elastic properties of the rocks. Cross plots analysis of different elastic parameters is generated to identify the lithology variability, pore-fluid type and to establish likely distinction between the hydrocarbon bearing sands, brine sands and shale. The study will help to better evaluate the hydrocarbon potential in the field. It will also help in an improved understanding of the physical properties of the reservoir and creating new exploration and development opportunities in the field.

## 2 Geological setting and petroleum system

**Fig. 1:**
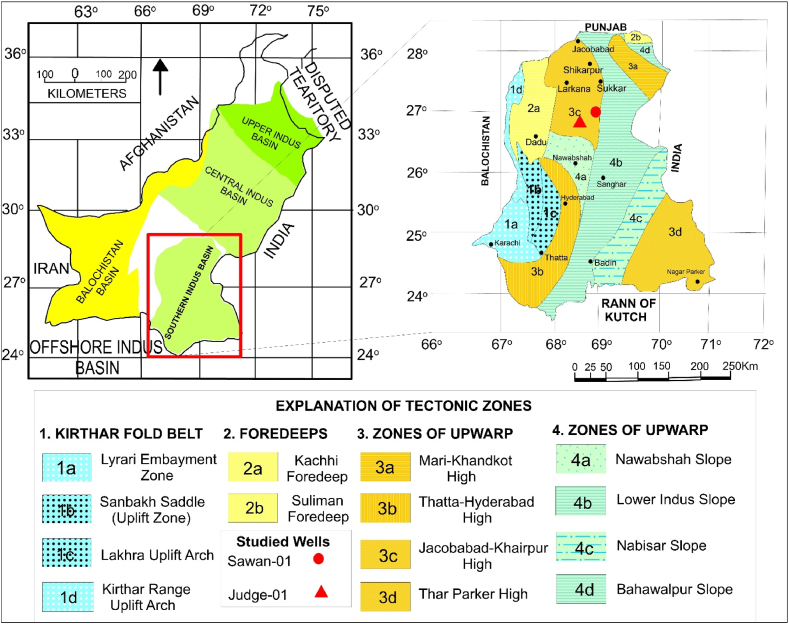
Geological and tectonic map of Southern Pakistan [17,18].

Tectonically, Pakistan is characterized on the basis of two convergent boundaries in the northeast, continent-island arc-continent collision boundary and southwest, Makran arc-trench gap and Afghan microplate obduction over the Arabian Plate [16]. The geological and tectonic map of Pakistan is shown in Fig. 1 [17,18]. The interest area lies in an extensional system with horst and graben structure and normal faulting. The rifted structure and faults are due to the divergence between the Indian Plate and the Gondwana supercontinent [5,[19], [20], [18]]. There are three post-rift tectonic events in the Indus Basin that are controlling the structural style of the basin. The first tectonic episode is uplifting during the Late Cretaceous epoch. In the second tectonic event during Eocene-Oligocene, most deep basement faults and shallow wrench faults are terminated against K/T unconformity formed due to erosion. The features formed during the cretaceous section are cut by NW-SE oriented wrench fault that changes the substantial single fault at Chilton top into multiple left lateral segments at Upper Goru and Lower Goru levels. In the third event of uplifting, from late tertiary to the present, Khairpur High was formed [3,5,21,22].

The Goru Formation acts as a hydrocarbon reservoir in the basin. It contains interbeds of siltstone and shale. The sandstone in the lower part of the formation has reservoir properties. Due to its reservoir rock properties, the LGF is a prime reservoir target in this basin for oil and gas companies. The formation is divided into various sequences with multiple sandstone packages. The shale beds present in the formation separate all the sandstone packages. This formation was deposited into the marine environment with deep water. The existence of oysters and pelecypods also indicates estuarine conditions for deposition [[23], [24], [25]].

**Fig. 2:**
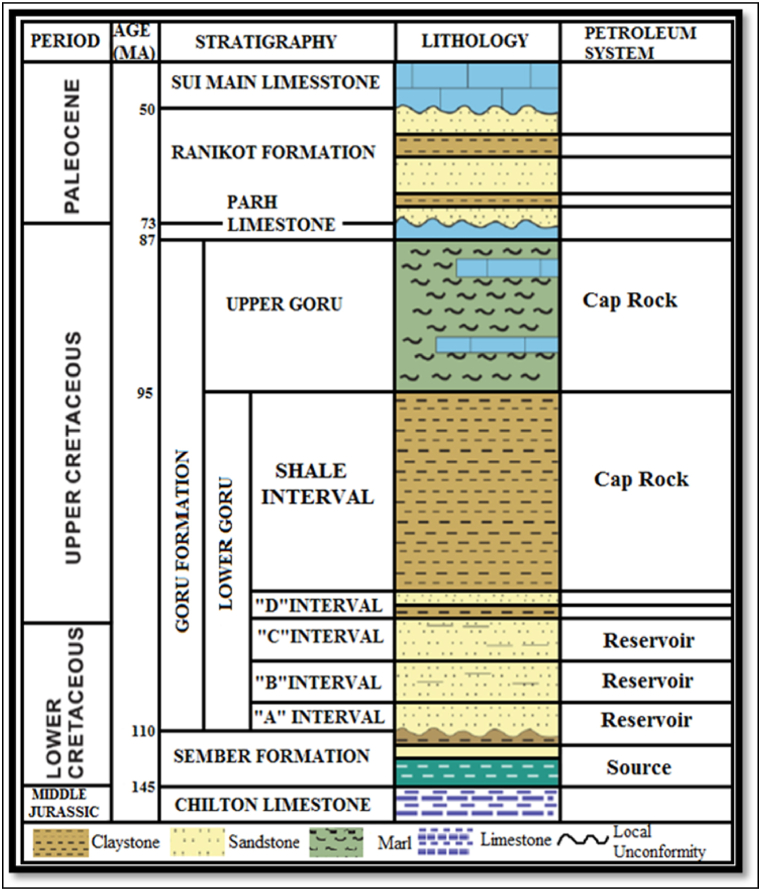
Generalized stratigraphy of the Sawan Gas Field [29,30].

The generalized stratigraphy of the Sawan Gas Field is shown in Fig. 2. There are two units of this formation named LGF and Upper Goru Formation. The Upper Goru Formation provides a seal for hydrocarbon accumulation in the basin. The nomenclature of the Lower Goru Formation (LGF) by different exploration and development companies is shown in Table 1 [26]. This Formation consists of shale and marl. It indicates high sand deposits with mixed carbonate clastic lithology. The lower sandstone in the formation is called A interval by OMV. At the time of the separation of the Indian Plate from Africa and the Antarctic Plates in Cretaceous, the marine basin was developed, and this interval was deposited in low stand sequence. The sandstone above the A interval is named as B interval and it is the main reservoir interval in the study area and the whole basin. This interval consists of sandstone with shale beds. Owing to the opening of the Indian Ocean during the Late Cretaceous, this interval was deposited in the transgression environment in low stand sequences. The C interval is also acting as a reservoir in the basin and wells are produced from this interval. This formation interval also consists of sandstone with shale beds. Shales of C interval are acting as a top seal for sandstone in the formation. The D interval is also called the Upper Sand interval and consists of sandstone with shale beds [21,27,28].

**Table 1:** Nomenclature of Lower Goru Formation by different exploration and development companies [26].

	OGDCL			UTP		LASMO	OMV
Lower Goru	Upper Sand	A Sand	Layer 1	Upper Sand	A Sand	Shale out in North	Shale out in North
		Turk Shale	Layer 2		Turk Shale		
		B Sand	Layer 3		B Sand		
		Badin Shale	Layer 4		Badin Shale		
		C Sand	Layer 5		C Sand		
		Jhol Shale			Jhol Shale		
		D Sand			D Sand		
	Upper Shale			Upper Shale		H Sand with Shale	No Significant Sand
	Middle Shale			Middle Shale		G Sand	D Sand
	Lower Shale			Lower Shale		F Sand with Shale	C Sand
	Basal Sand			Basal Sand		E Sand	B Sand
	Talhar Shale			Talhar Shale		C & D Sand within Shale	
	Massive Sand			Massive Sand		B Sand	A Sand

The most reservoir rocks in the LIB lie within the Cretaceous strata. The Lower Goru Formation in the study area has good reservoir characteristics. All the hydrocarbon potential of Sindh Monocline is present in LGF. The B and C intervals of the formation are the primary reservoir rocks in this area. The depositional environment for the formation is barrier bar and lower shore facies to deltaic. The benthonic fauna also indicates shallow marine at some places [16,31].

Due to the organic presence and thermal maturity of Lower Cretaceous Sembar Formation shale unit, it is best to source rock in the study area that can generate the gas. The determination of oil and gas in this formation indicates a reducing environment for the formation. This formation is prolific for gas due to the richness of type III kerogen. The sandstone in this formation has characteristics of a potential reservoir [5,16,31].

The tectonic events in the Lower Indus Basin are responsible for normal faulting and horst and graben structures. The Khairpur high formed the structural traps in the study area [20,24]. These faults provide the seal for hydrocarbon accumulation. The main seal rocks are thick deposits of marl and shale in the Upper Goru Formation. The shales inside Lower Goru Formation also act as a seal in this area [7,16].

## 3 Methods and database

Current research of the Lower Goru Formation included seismic structural interpretation and attributes and well logs interpretation for lithology and mineral identification, petrophysical analysis, rock physics analysis and fluid substitution.

**Fig. 3:**
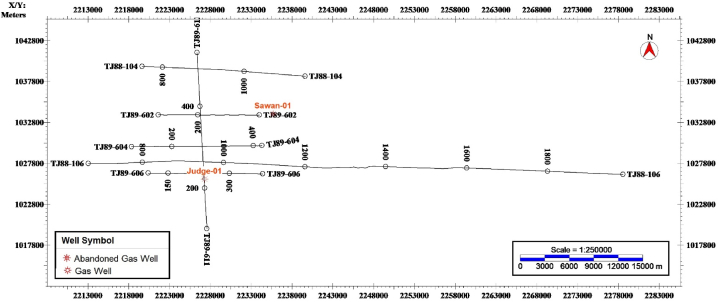
Base map of the study area showing the seismic lines and wells.

The wireline logs of Judge-01 well located at Latitude of 26° 54' 50.4″N and Longitude of 68° 47' 51.7″E and Sawan-01 well located at Latitude 26° 59' 30.58″N and Longitude of 68° 54' 25.17″E were used in this study. The wireline logs of Gamma Ray, Calliper, Resistivity, PEF, Sonic, Neutron, and Density were used for petrophysical analysis. In addition, the formation tops in the borehole of these wells were also used in the petrophysical analysis and structural interpretation. For structural interpretation and reservoir characterization, seismic lines were utilized in digital format (SEG-Y). These seismic profiles were loaded in Kingdom suite software and the generated base map shown in Fig. 3 displays the location of lines concerning wells.

### 3.1 Limitation of the study

2D seismic data set has limited spatial resolution, azimuthal coverage, frequency content, and data volume. Despite these limitations, 2D seismic data can still provide valuable insights into the subsurface when used in conjunction with other data sets, such as well logs and geological maps. One limitation is that 2D seismic data only provides a single cross-sectional view of the subsurface, whereas 3D seismic data provides a much more comprehensive view of the subsurface. This means that some seismic attributes that rely on 3D information, such as curvature or dip, may not be as reliable when computed from 2D seismic data [32,33]. However, it is important to be aware of these limitations and to carefully evaluate the quality and reliability of any attribute results obtained from 2D seismic data and local calibration of geological data set. Additionally, some seismic attributes are less affected by the limitations of 2D seismic data, such as acoustic impedance and Poisson's ratio.

### 3.2 Seismic structural interpretation and attributes analysis

Reflectors were identified on the basis of prominent adjacency of reflectors on seismic sections [34]. The seismic structural interpretation includes different steps i.e., well to seismic tie, horizon picking, fault marking, velocity analysis, time, and depth contouring. The literature review shows that because normal faulting, horst, and graben structure and negative flower structure are dominant in this area. Therefore, structural interpretation was performed to understand the subsurface structure. The first and most important step in seismic interpretation is well to seismic tie. There are several ways to do it, but the most practiced methods are through the generation of the synthetic seismogram.

**Fig. 4:**
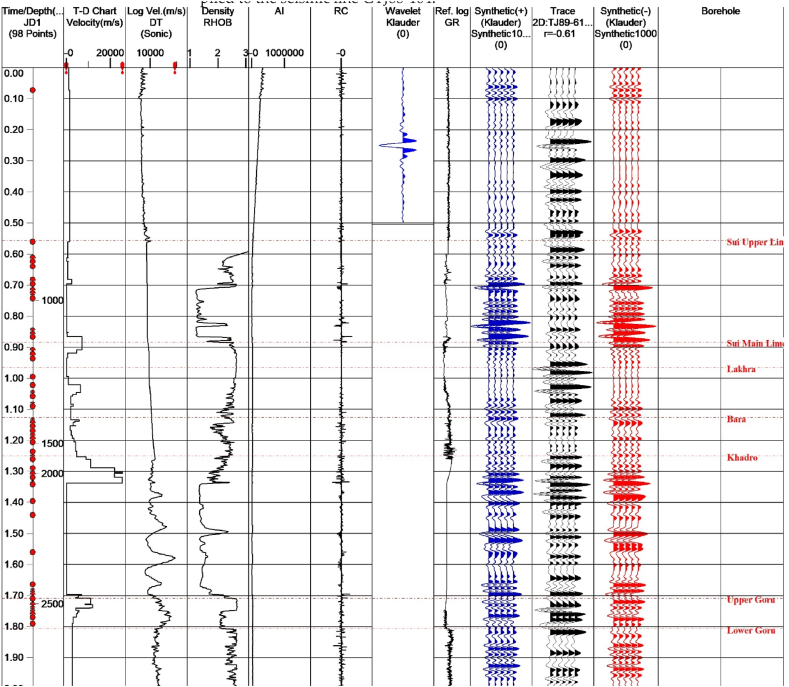
Synthetic seismogram of Judge-01 well.

A velocity panel of the nearest SP from Judge-01well on the TJ89-611 seismic line was used to generate a time-depth relationship. The sonic log was used for velocities calculation and density log to get densities. The Klauder wavelet was used for this intended purpose. An appropriate trace was extracted from seismic data and a synthetic seismogram was generated as shown in Fig. 4. The seismic line GTJ89-611 was selected as a control line because it crossed all other dip lines. The well was used to generate a synthetic seismogram was located on this line. The horizons were first marked on this line through formation tops by using a synthetic seismogram. The marked horizons are Lower Goru top, Upper Goru top, Sui Main Limestone top and Sui Upper Limestone top. The B sand top, C sand top and Chilton top are marked randomly because their tops were not present. The identified horizons on the GTJ89-611 seismic line was marked on dip lines after miss tie calculation. The misties were present in the given data due to different data vintages. These mis-ties were removed from the data by comparing it with the GTJ89-611 seismic line as standard. The data was from different vintages; therefore, phase rotation was applied to the seismic line GTJ88-104.

### 3.3 Petrophysical analysis

Several processes were performed for petrophysical analysis. They started by marking the possible reservoir zone. Then the volume of shale and porosities were calculated. The resistivity of water was calculated for the estimation of water and hydrocarbon saturation. Different cross plots were formed and finally, lithology was identified. The crossover of neutron and density log curves was a reliable hydrocarbon zone indicator. The gamma ray index was calculated in the first step using equation (1) to estimate the volume of shale.

(1) $$I_{GR}=\frac{GR_{log}-GR_{\min}}{GR_{\max}-GR_{\min}}$$

where $I_{GR}$ represents volume of shale index, $GR_{log}$ indicate log value at zone, $GR_{\min}$ is log value at the clean zone and $GR_{\max}$ indicates maximum gamma ray value. The Stieber equation gives a better estimation of shale volume in the gas reservoir [28]. As we know, the Lower Goru Formation is gas-producing in the study area. So, we used Stieber eq. (2) for shale volume calculation [35].

(2) $$V_{sh}=\frac{I_{GR}}{3-2I_{GR}}$$

where $V_{sh}$ indicates the shale volume.

The rock porosity can be calculated by using multiple techniques. However, acoustic log, neutron log, and density logs were mainly used for this purpose [36]. The sonic porosity was measured with the help of Wyllie time average eq. (3).

(3) $$\varphi_{S}=\frac{\Delta t_{\log}-\Delta t_{m}}{\Delta t_{f}-\Delta t_{m}}$$

where, $\varphi_{S}$ = sonic derived porosity, $\Delta t_{m}$ = matrix transit time that is 55 μs/feet, $\Delta t_{f}$ = fluid transit time that is 185 μs/feet in this case and $\Delta t_{\log}$ = sonic transit time from log

The density log was used primarily as porosity logs. The porosity from the density log was calculated by using eq. (4) [36].

(4) $$\varphi_{D}=\frac{\rho_{ma}-\rho_{b}}{\rho_{ma}-\rho_{f}}$$

where, $\varphi_{D}$ = density derived porosity, $\rho_{ma}$ = matrix density, $\rho_{f}$ = fluid density and

$\rho_{b}$ = bulk density values from the log

The neutron log computes the amount of hydrogen atom in a rock. So, it gives the liquid-filled porosity of the rock. It is primarily used for the identification of porous formation. The neutron log readings are basically the neutron porosity [36,37].

The average porosity is calculated using eq. (5) of the average neutron density porosity [38].

(5) $${\varphi_{N}}_{D}=\sqrt{\frac{{\varphi_{N}}^{2}+{\varphi_{D}}^{2}}{2}}$$

where ${\varphi_{N}}_{D}$ is average porosity, $\varphi_{N}$ is neutron porosity and $\varphi_{D}$ indicates density porosity.

The effective porosity ($\varphi_{E}$) is the number of connected pores in a rock. It was calculated by using eq. (6) [36,37].

(6) $$\varphi_{E}=\varphi_{ND}\left(1-V_{sh}\right)$$

The resistivity of mud filtrate was calculated by using the Gen-6 chart from the Schlumberger chartbook. We calculated the $R_{mfeq}$ by using the SP-2 chart and it was 0.028 Ω-m. After that, we calculated the static spontaneous potential by using the SP log. The difference between the shale baseline and sand baseline is +20 mV. Next, we plotted the value of SSP and $R_{mfeq}$ on the SP-1 chart and calculated the $R_{weq}$ value. The chart reading for $R_{weq}$ was 0.043 Ω-m. After that, the $R_{weq}$ value was used to find out $R_{w}$ by using the SP-2 chart. The $R_{w}$ was an estimated 0.041 Ω-m. Similarly, $R_{w}$ was calculated for the remaining zones of Sawan-01 well and Judge-01.

Water saturation is determined by using Archie's equation [39] (eq. (7)).

(7) $$S_{w}=\sqrt{F\frac{R_{w}}{R_{t}}}$$

where $S_{w}$ is water saturation, $R_{w}$ is the resistivity of formation water, F is formation factor and $R_{t}$ is transition zone resistivity.

The saturation of hydrocarbon is computed by utilizing eq. (8).

(8) $$S_{h}=1-S_{w}$$

where $S_{h}$ indicates saturation of hydrocarbon and represents the water saturation.

### 3.4 Rock properties

The bulk modulus (K) of a rock is a ratio of hydrostatic stress to volumetric strain. It can be calculated by both laboratory measurements of velocities and wireline log analysis. K was calculated using eq. (9) [40].

(9) $$K=\rho_{b}\left(V_{p}^{2}-\frac{4}{3}V_{s}^{2}\right)$$

where, $V_{p}$ is p wave velocity, K is the bulk modulus, $V_{s}$ is secondary wave velocity and $\rho_{b}$ is bulk density.

The shear modulus (G) is the ratio of shear stress to shear strain [40]. It also can be determined by using eq. (10) [40].

(10) $$G=\rho_{b}*V_{s}^{2}$$

where, $V_{s}$ is shear wave velocity, $\rho_{b}$ is bulk density and G is the shear modulus. The bulk density is also an important parameter in rock physics analysis. The saturated density ($\rho_{sat}$) was calculated for the desired saturation level by using eq. (11) [40].

(11) $$\rho_{sat}=\varphi \rho_{fl}+\rho_{mat}\left(1-\varphi \right)$$

### 3.5 Fluid substitution

The body wave velocities and densities are primary parameters that control the seismic response of a reservoir. The parameters that were used in the study are given in Table 2. The P wave velocity was obtained from the DT log and secondary wave velocity was calculated from primary wave velocity utilizing the forward method. The acoustic impedance and shear impedance were calculated by multiplying seismic velocities and density. After computing all the necessary parameters their cross plot was formed for rock physics analysis. For initial in-situ saturation of fluids, the p wave velocity was calculated from a sonic log using eq. (12).

(12) $$V_{p}=\frac{1}{DT}$$

where, $V_{p}$ is p wave velocity and DT is sonic log.

**Table 2:** Parameters used in Gassmann fluid substitution modified after [13].

Parameters	Symbols	Values	Units
Bulk modulus of quartz	K_qtz_	37	GPa
Shear modulus of quartz	U_qtz_	45	GPa
Density of quartz	$\rho_{qtz}$	2.65	g/cc
Bulk modulus of shale	K_shl_	25	GPa
Shear modulus of shale	U_shl_	10	GPa
Density of shale	$\rho_{shl}$	2.55	g/cc
Bulk modulus of gas	K_g_	0.0797	GPa
Density of gas	$\rho_{g}$	0.1712	g/cc
Bulk modulus of brine	K_b_	3.17	GPa
Density of brine	$\rho_{b}$	0.948	g/cc
Specific gravity of gas	G_gas_	0.642	SG
Salinity of brine	S_b_	20000	ppm
Reservoir temperature	T	175	^0^C
Reservoir pressure	P	37.14	MPa

Shear wave velocity was calculated at the initial in-situ saturation level. The Castagna equation (13) was used for this purpose [41].

(13) $$V_{p}=1.16V_{s}+1.36$$

where, Vp is Primary wave velocity and Vs is secondary wave velocity. The density at initial level of saturation is obtained simply from density log curve.

Gassmann eq. (14) is commonly used for fluid substitution. It correlates the saturated bulk modulus of rocks to its bulk modulus of the dry frame, bulk modulus of fluid, bulk modulus of matrix and porosity [13,40,42].

(14) $$K_{sat}=K_{dry}+\frac{{\left(1-\frac{K_{dry}}{K_{mat}}\right)}^{2}}{\left[\frac{\varphi }{K_{fl}}+\frac{\left(1-\varphi \right)}{K_{mat}}-\frac{K_{dry}}{K_{mat}^{2}}\right]}$$

where, $K_{dry}$, $K_{mat}$, $K_{sat}$ and $K_{fl}$ are dry, matrix, saturated, fluid bulk modulus respectively, and φ is porosity.

The bulk modulus of the matrix is the combined modulus of individual minerals in the rock. The Reuss-Hill averaging eq. (15) was applied to measure the bulk modulus of the matrix [43].

(15) $$K_{mat}=\frac{1}{2}\left[\left(V_{clay}K_{clay}+V_{qtz}K_{qtz}\right)+\left(\frac{V_{clay}}{K_{clay}}+\frac{V_{qtz}}{K_{qtz}}\right)\right]$$

Where, $V_{clay}$ is clay volume, $V_{qtz}$ is the volume of quartz, $K_{clay}$ is the bulk modulus of clay and $K_{qtz}$ the bulk modulus of quartz. The quartz and clay are considered as a mineral matrix because the reservoir is in Lower Goru Formation. The volume of quartz and clay were secured from the petrophysical analysis. The values $K_{clay}$ and $K_{qtz}$ were obtained from Ref. [13]. The matrix density ($\rho_{mat}$) was calculated by using eq. (16).

(16) $$\rho_{mat}=V_{clay}\rho_{clay}+V_{qtz}\rho_{qtz}$$

The bulk modulus of the dry frame is compulsory for Gassmann fluid substitution. It was used as a constant in fluid substitution. It was calculated using eq. (17) by Ref. [44].

(17) $$K_{dry}=\frac{K_{sat}\left(\frac{\varphi K_{mat}}{K_{fl}}+1-\varphi \right)-K_{mat}}{\frac{\varphi K_{mat}}{K_{fl}}+\frac{K_{sat}}{K_{mat}}-1-\varphi }$$

where, $K_{dry}$, $K_{mat}$, $K_{fl}$, and $K_{sat}$ are dry, matrix, fluid, and saturated bulk modulus and φ is porosity.

The fluid properties, i.e., bulk modulus and density, are also compulsory for fluid substitution. The fluid properties are calculated by using the empirical equations given by Batzle and Wang. These parameters are required for both in-situ and substituting fluids [40,45]. The fluid's bulk modulus for a two-component gas-water system can be computed by utilizing eq. (18) [40,46].

(18) $$\frac{1}{K_{fl}}=\frac{S_{w}}{K_{w}}+\frac{S_{h}}{K_{h}}$$

where, $K_{fl}$ is fluid's bulk modulus, $K_{w}$ is the bulk modulus of water, $S_{w}$ is water saturation, $S_{h}$ is hydrocarbon saturation and $K_{h}$ is the bulk modulus of hydrocarbon. The density of fluid utilized eq. (19).

(19) $$\rho_{fl}=S_{w}\rho_{w}+S_{h}\rho_{h}$$

where, $S_{w}$ is water saturation, $S_{h}$ is hydrocarbon saturation, $\rho_{fl}$ is the fluid density, $\rho_{h}$ is hydrocarbon density.

## 4 Results and discussions

### 4.1 Seismic structure interpretation

The observed faults are normal faults with NW-SE trends. The seismic lines indicate that normal faults show two different behaviors i.e., minor fault and major fault. The major faults are very high dip angle faults while minor faults are branches of the main fault. The minor faults have an enormous throw and major faults are penetrating deeper than minor faults. Mostly minor faults are responsible for negative flower structure [19,47,48].

The alluvium is the shallowest and Chilton Formation top is the deepest horizon that is encountered in the well which indicates in the interpreted seismic lines. Owing to strong acoustic impedance contrast, the reflectors of top of Lower Goru Formation, top of B Sand and top of Sui Main Limestone are stronger than other horizons. The high contrast at tops is due to change in lithology from marl beds in Upper Goru to sandstone in Lower Goru Formation and B Sand. The faults are marked based on discontinuity in seismic continuous reflections.

**Fig. 5:**
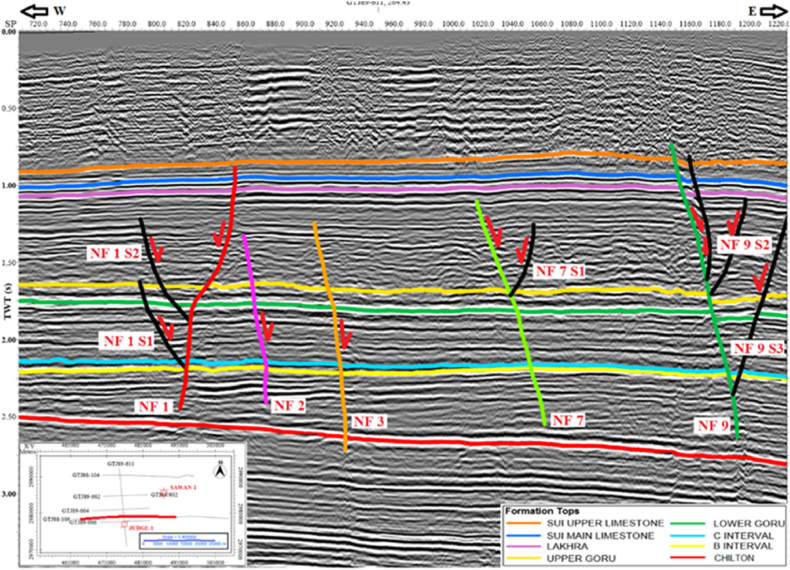
Interpreted seismic section GTJ88-106.

GTJ88-106 is a dip line and its orientation is west-east direction. The horizons are dipping in the east direction. The horst and graben structures are identified in both east and west directions. All the identified faults are normal fault and most of them are deep-rooted. One fault NF 1, on the west side of the line is dipping in SW direction and all other faults are dipping in SE direction (Fig. 5).

### 4.2 Seismic attributes

**Fig. 6:**
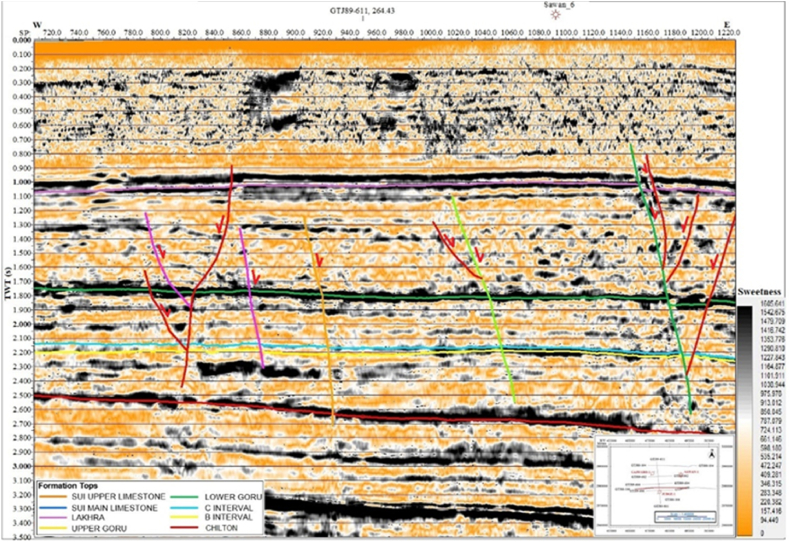
The interpreted seismic line GTJ88-106 (Sweetness).

Multiple structural attributes were applied to study the structural variations in the geological Formation [47]. The sweetness attribute is designed to identify the hydrocarbon prone places. These hydrocarbon places are also called sweet spots. The attribute is obtained by dividing the trace envelope attribute by average frequency. The hydrocarbon spots are indicated by high sweetness anomaly [49]. It also improves the imaging of sandstone rock bodies. In the study area, the sweetness attribute shows a high sweetness anomaly on seismic line GTJ88-106 between the SP 1020 and SP 1180 at B sand and C sand level as shown in Fig. 6. These high sweetness anomalies may indicate the presence of hydrocarbon at that place.

**Fig. 7:**
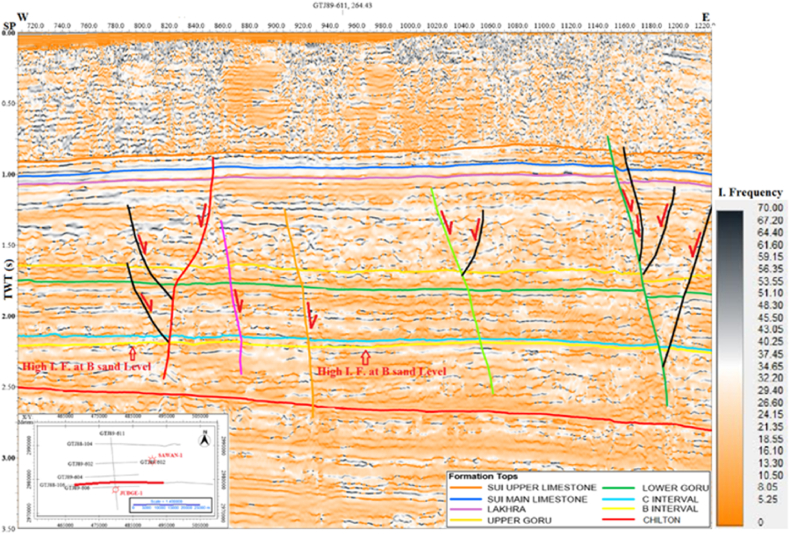
Interpreted seismic line GTJ88-106 (Instantaneous frequency).

In this study, the instantaneous frequency attribute results are not good enough for hydrocarbon identification. However, this attribute gives good results for lateral changes in lithology thickness. There are not much frequency variations at different horizons levels as shown in Fig. 7. There is a small increment in frequency at B sand level indicating small shale beds in sandstone lithology.

**Fig. 8:**
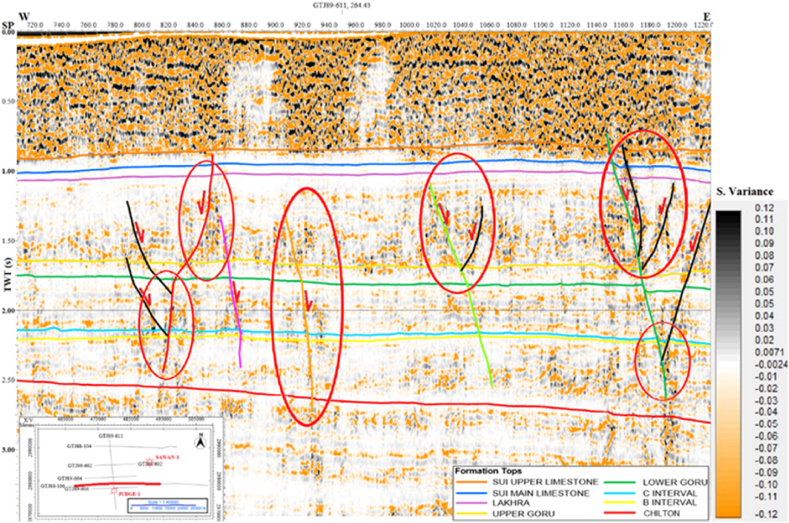
Interpreted seismic line GTJ88-106 (Similarity variance).

The similarity variance attribute also shows normal faulting with negative flower structure in the study area as shown in Fig. 8. The selected closure at B sand and C sand level are verifying the structure favorable for hydrocarbon near NF7. The Judge-01 well is drilled near closure formed at NF3.

### 4.3 Depth contour maps

**Fig. 9:**
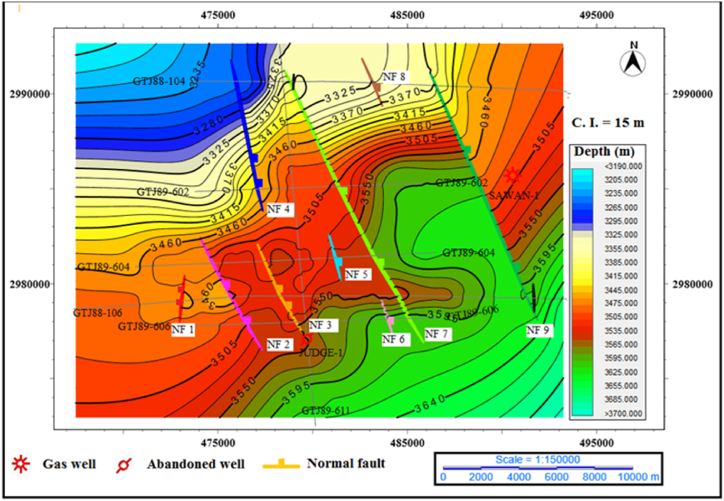
The depth contour map of the top of B interval.

TWT maps of top of B Interval: The depth contour maps of B interval top confirm the extensional regime in this area. The horst and graben structures are clearly visible in the contour map. The contour values are increasing in the south-east direction and confirm the structural trend of the area. The contours are closing near the NF 7, NF 2 and NF 3 faults at the central part of the map and represent the trapping structures for hydrocarbon accumulation, as shown in Fig. 9.

**Fig. 10:**
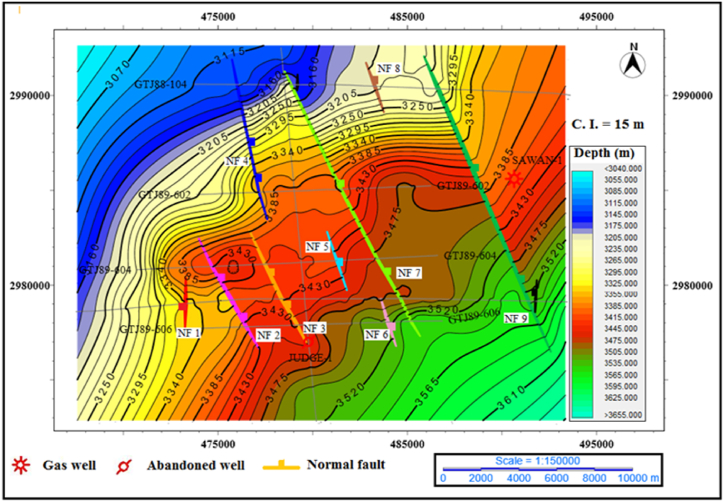
The depth contour map of the top of C interval.

Depth maps of top of C Interval: The C interval is a reservoir in this area, and it is indicating a closure near Sawan-1 well but due to data limitation, it is not clear here. The depth contour is showing normal faulting in this area. The contours are closing near the fault NF 7 forming the horst and graben structures. The contours are striking in the NE-SW direction. The depth contour value is low on the north-west side and high on the south-east side. The extensional faults that are generating horst and graben structures are striking in the NW-SE direction. The Judge-1 well is drilled at horst location as shown in Fig. 10.

**Fig. 11:**
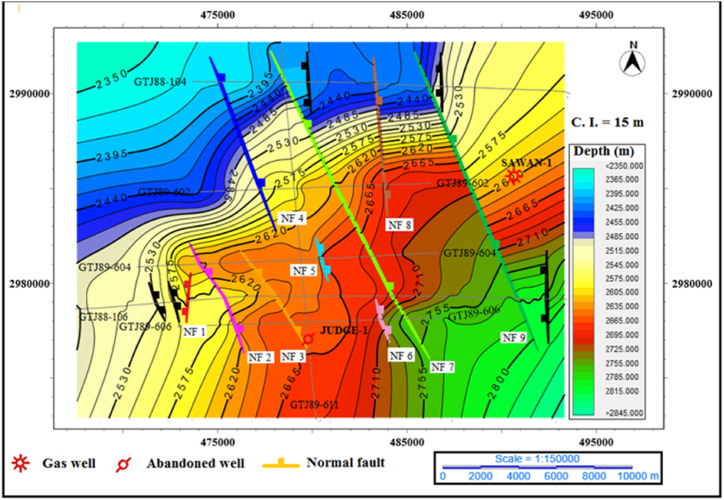
The depth contour map of the top of Lower Goru Formation.

**Depth map of top of Lower Goru Formation**: The depth contour map of top of Lower, Goru Formation are shallowest on the north-west side. The contours trend is NE-SW, and it changes to E-W near the NF7 and NF9 showing local anomalies. The minimum depth contour value is 2365 m on seismic line GTJ88-104. The normal fault system is intercepting the structure in the NW-SE direction. This extensional fault system is generating horst and graben structure as shown in Fig. 11.

### 4.4 Petrophysical interpretation

**Fig. 12:**
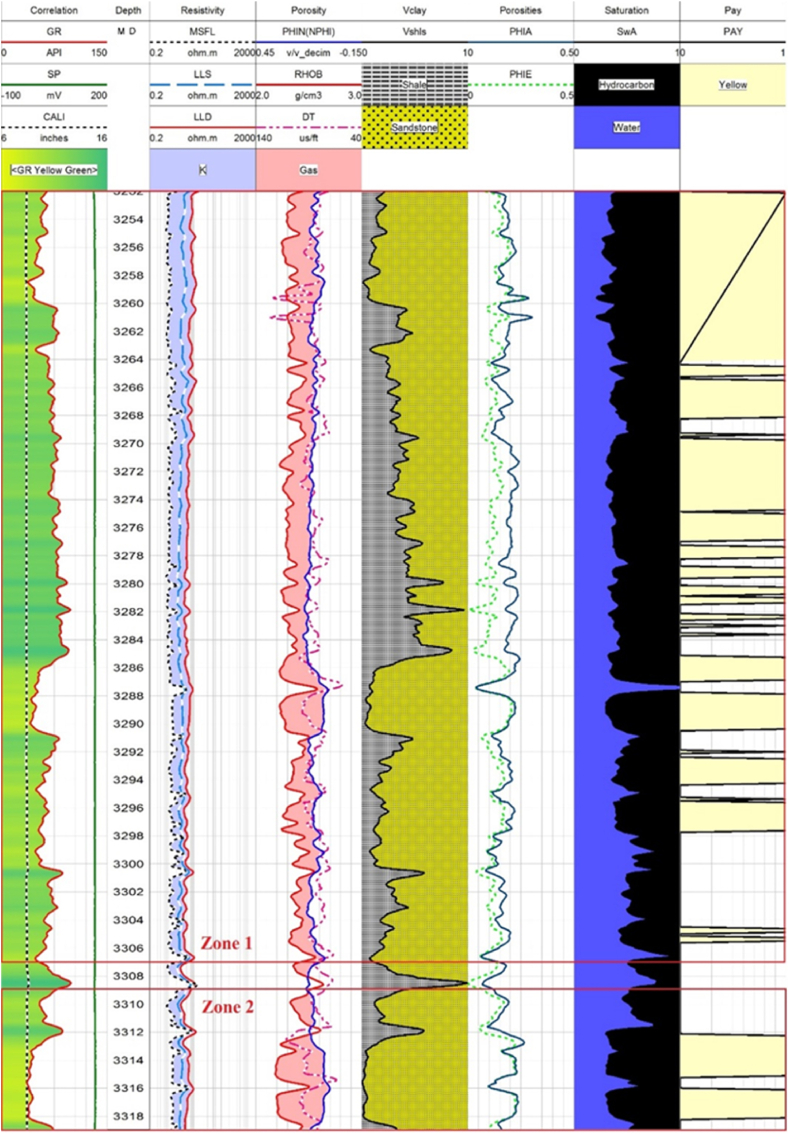
The log response in Zone 1 and Zone 2.

Sawan-01 well: The caliper log curve shows that the borehole condition is very good. The depth of the possible reservoir zone is ranging from 3252 to 3320 m. This zone is further divided into 4 parts based of log analysis. The average values of water saturation and hydrocarbon saturation indicates that only zone 1 and zone 2 have good potential for hydrocarbon, as shown in Fig. 12.

**Fig. 13:**
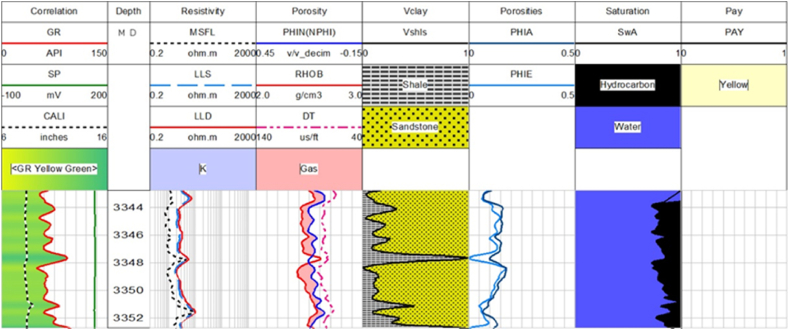
The log response in zone 3.

**Fig. 14:**
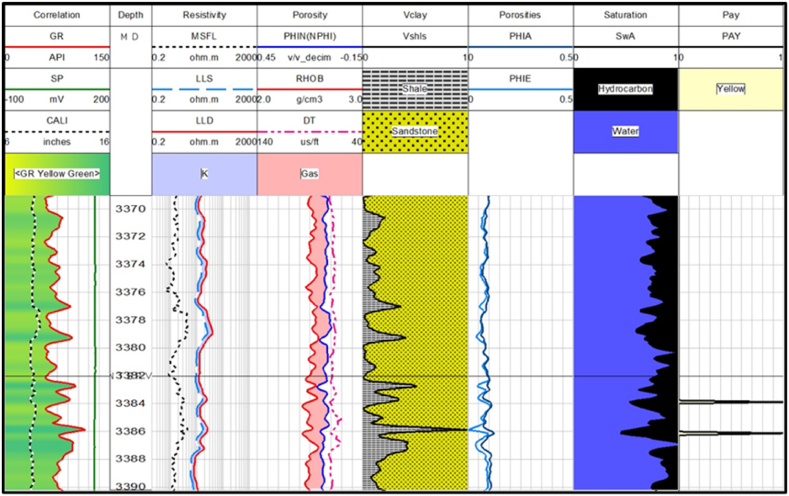
The log response in zone 4.

The average values of hydrocarbon saturation are very low in zone 3 and zone 4 as shown in Fig. 13, Fig. 14. Though the volume of sandstone is greater in these zones, effective and average porosities are very low. Therefore, it can be concluded that zone 3 and zone 4 are less favorable for hydrocarbon potential than zone 1 and zone 2. The petrophysical interpretation results of Sawan-01 well in Table 3.

**Table 3:** The petrophysical interpretation results of Sawan-01 well.

Depth(m)	$V_{Sh}$ (%)	$\Phi_{D}$ (%)	$\Phi_{N}$ (%)	$\Phi_{S}$ (%)	$\Phi_{A}$ (%)	$\Phi_{E}$ (%)	$S_{W}$ (%)	$S_{H}$ (%)
3252–3307	27.5	17.72	12.23	22.36	17.45	12.53	39	61
3309–3319	12.50	19.34	9.15	25.81	18.07	16.03	42	58
3342.8–3352.3	19.6	11.73	11.86	15.39	12.99	10.5	83.39	16.61
3369–3390	14.7	10.26	6.28	9.94	8.83	7.54	76.64	33.36

**Fig. 15:**
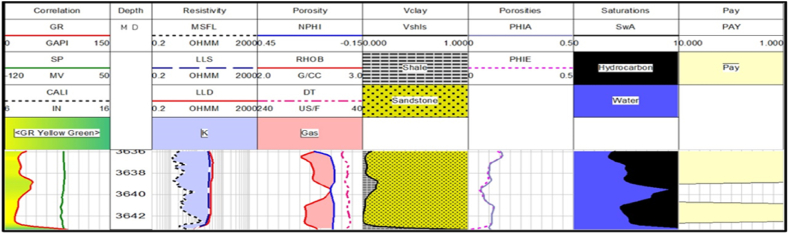
The log response in zone 1.

**Fig. 16:**
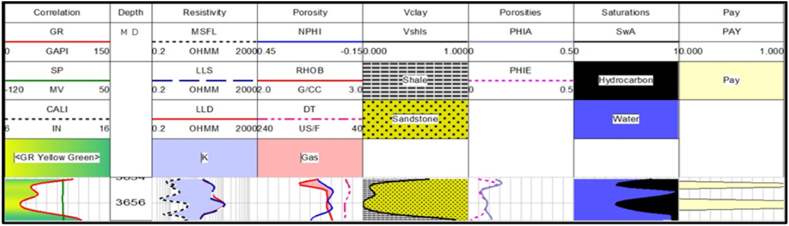
The response of log in zone 2.

**Judge -01 well**: There is only two possible reservoir zone in this well. The thicknesses of possible reservoir zones are very small. The interpretation results show that only zone 1 has hydrocarbon potential as shown in Fig. 15. The average hydrocarbon values in zone 2 are very low. Therefore, it does not hold hydrocarbon potential as shown in Fig. 16. The average percentage of all the measured parameters is given in Table 4.

**Table 4:** The petrophysical interpretation results of Judge-01 well.

Depth(m)	$V_{Sh}$ (%)	$\Phi_{D}$ (%)	$\Phi_{N}$ (%)	$\Phi_{S}$ (%)	$\Phi_{A}$ (%)	$\Phi_{E}$ (%)	$S_{W}$ (%)	$S_{H}$ (%)
3635.80–3643.27	7.94	13.46	2.54	13.51	9.84	9.28	52	48
3654.9–3657.45	19	3.38	5.93	17.09	8.63	6.73	73.33	26.66

### 4.5 Lithology identification

Lithology identification is necessary for the estimation of correct porosity values. The neutron and density log values do not depend only on porosities but also on lithology. Therefore, these log values can be utilized for lithology identification. The three solid lines on neutron and bulk density cross plot represent the pure lithology of sandstone, limestone, and dolomite in ideal condition. If the data points fall precisely on these lines, then the lithology is pure. However, if data point falls between solid lines, they represent the mixed lithology [36].

**Fig. 17:**
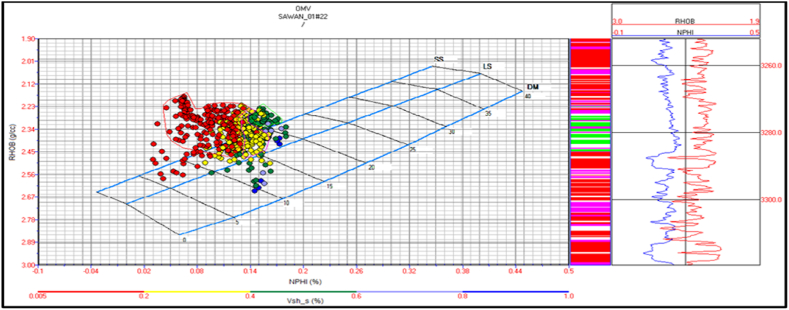
The lithology cross plot of zone 1 and 2 in Sawan-01 well.

Sawan-01 Lithology: There is four identified zone in this well. The cross plot of zone 1 and zone 2 indicate that lithology is mainly sandstone. The central part of the zone at a depth of 3280 m indicates shaly sandstone as shown in Fig. 17.

**Fig. 18:**
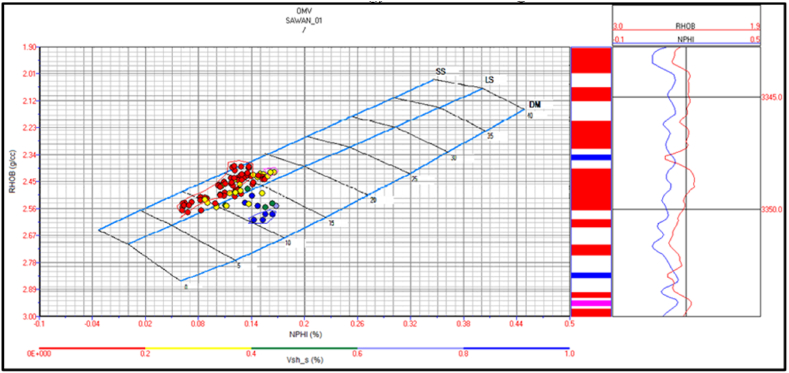
The lithology cross plot of zone 3 in Sawan-01 well.

**Fig. 19:**
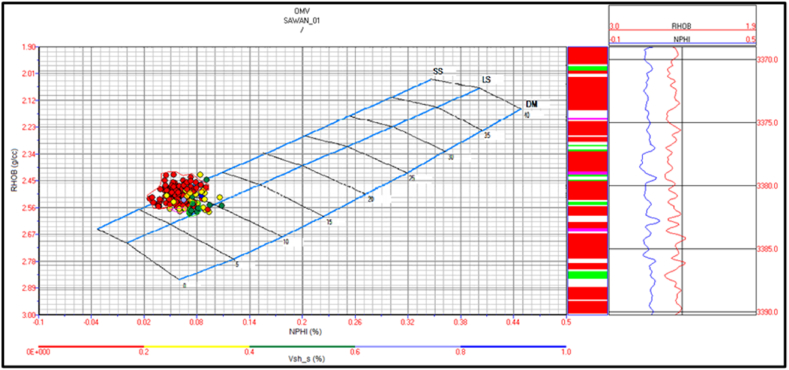
The lithology cross plot of zone 4 in Sawan-01 well.

The cross plot for zone 3 indicates sandstone lithology with a minor shale percentage. There are few packages of shaly limestone between sandstone beds as shown in Fig. 18. Zone 4 indicates sandstone lithology, as shown in Fig. 19.

### 4.6 Mineral identification

The identification of mineral is essential to measure rock properties like density. The density porosity is an essential parameter in petrophysical analysis for reservoir characterization. So, mineral identification was done to measure the actual density of the rocks. The potassium, thorium and PEF logs are used for the identification of clay type. The type of clay reflects the origin of rock [15,50]. The origin of a rock describes the rock properties more precisely.

**Fig. 20:**
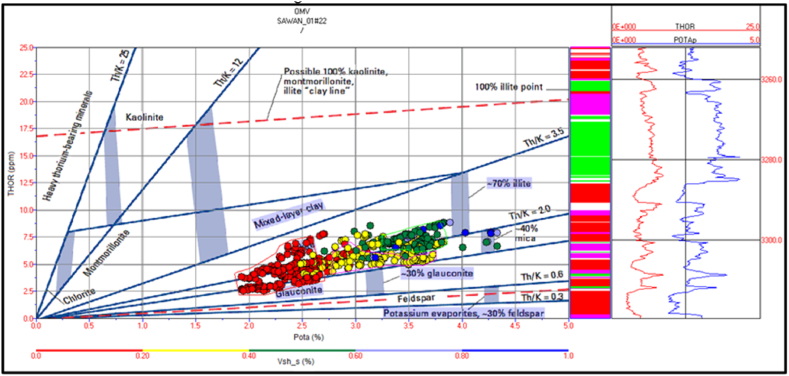
The potassium vs. thorium cross plot of zone 1 and 2 in Sawan-01 well.

**Fig. 21:**
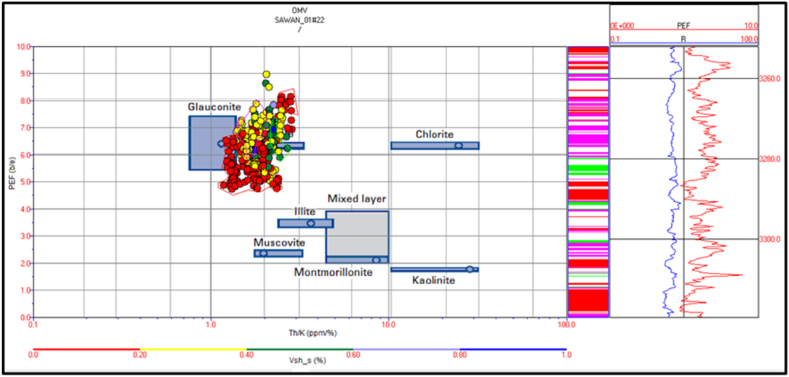
The PEF vs. thorium/potassium cross plot of zone 1 and 2 in Sawan-01 well.

The spectral gamma ray logs are missing in Judge-01 well. Therefore, mineral identification in Judge-01 well is not possible for this study. The potassium versus thorium cross plot of zone 1 and 2 in Sawan-01 well indicate that illite and mica clay are dominant minerals. Therefore, the sandstone is micaceous in this zone. The shaliness of the formation is decreasing with increasing depth, as shown in Fig. 20. The cross plot of PEF versus thorium/potassium also confirms the results of potassium vs. thorium cross plot for this zone, as shown in Fig. 21.

**Fig. 22:**
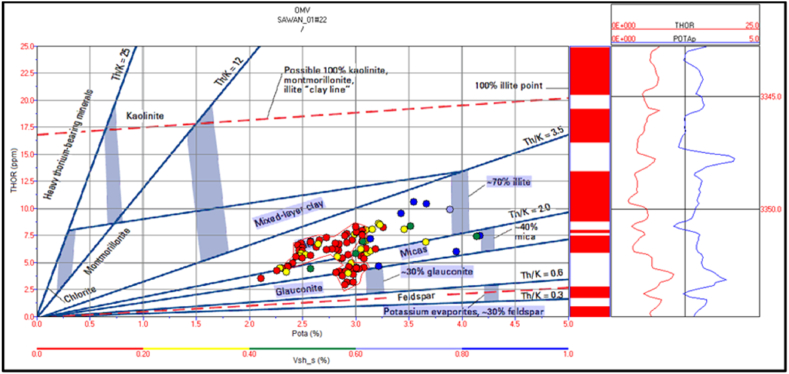
The potassium vs. thorium cross plot of zone 3 in Sawan-01 well.

**Fig. 23:**
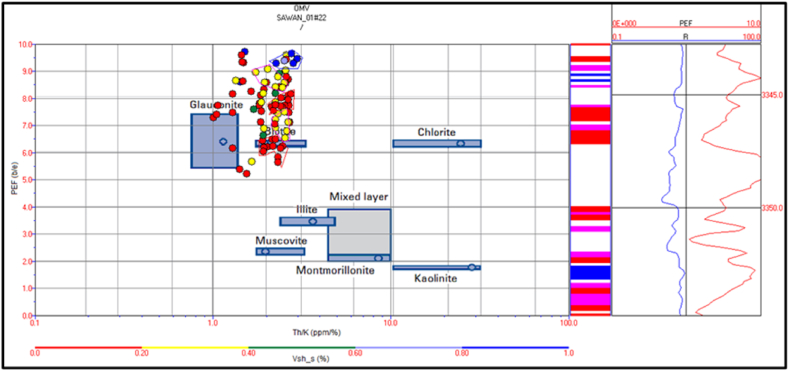
The PEF vs. thorium/potassium cross plot of zone 3 in Sawan-01 well.

The cross plot of potassium vs. thorium in zone 3 depicts the mixed clay layer including mica, illite and, glauconite. Therefore, the sand is greensand and micaceous sand with some illite concentration. The shale percentage in this zone is less (>20%) shown by red color circles in Fig. 22. The PEF vs. thorium/potassium ratio cross plot also verifies this result as shown in Fig. 23.

**Fig. 24:**
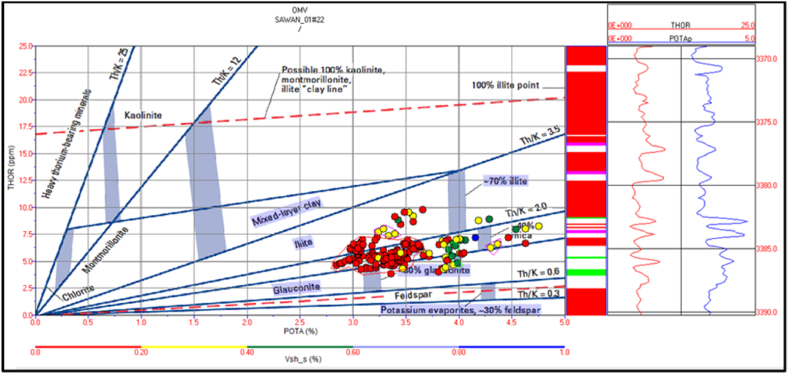
The potassium vs. thorium cross plot of zone 4 in Sawan-01 well.

**Fig. 25:**
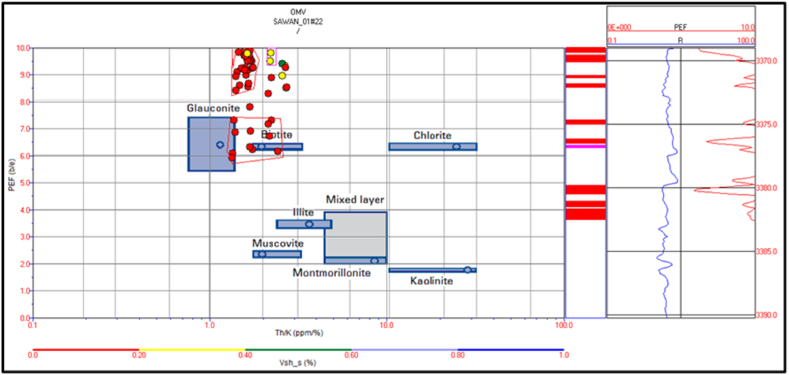
The PEF vs. thorium/potassium cross plot of zone 4 in Sawan-01 well.

Zone 4 depicts a similar clay type as in zone 3 but with a higher percentage of minerals. The sandstone is micaceous and green sand with some illite portion. The shale percentage increases with depth as shown in Fig. 24. The PEF vs. thorium/potassium cross plot also shows similar results for this zone as shown in Fig. 25.

The primary lithology is sandstone in the Sawan-01 well. The mineral composition of the C sand also confirms the reservoir characteristics like the depositional environment and rock type. Therefore, C sand in this well is a good reservoir. Zone 1 and zone 2 have up to 15% porosity and 52% hydrocarbon saturation. Therefore, zone 1 and zone 2 has good hydrocarbon potential in this well. The other zones have a low percentage of hydrocarbon saturation and porosities. So, they have low hydrocarbon potential in Sawan-01 well.

The petrophysical analysis of Judge-01 well indicates sandstone as primary lithology. There are only two possible reservoir zones in this well. Zone 1 with 48% hydrocarbon saturation and 9.28% effective porosity have hydrocarbon potential.

### 4.7 Results of fluid substitution

The Gassmann fluid substitution was applied to the well data of Sawan-01 and Judge-01 at already identified prospective zones of B sand and C sand. The fluid substitution was applied in three zones in Sawan-01.

**Fig. 26:**
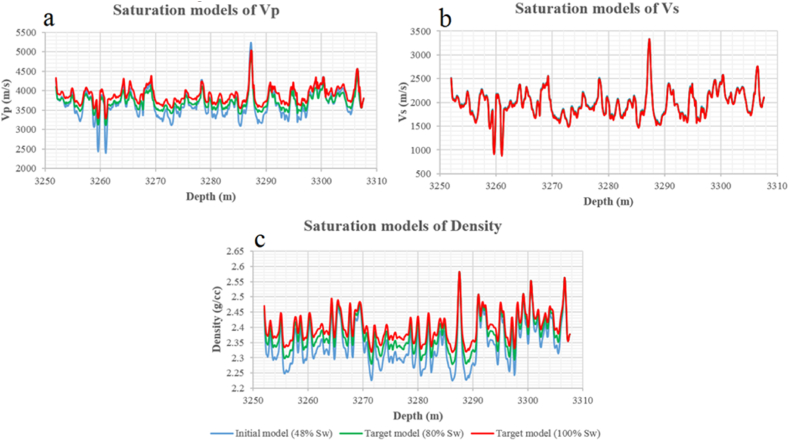
The variations of seismic velocities and density at 80% and 100% Sw level after Gassmann fluid substitution: (a) P wave velocity, (b) S wave velocity, (c) Saturated density.

**Sawan-01:** The initial water saturation is 48% in zone 1 (3252–3307 m) obtained from the petrophysical analysis. The target saturation level is 80% and 100% water saturation in this zone. The p-wave velocity increases with an increase in water saturation level from initial to 80% and 100% saturation level in all zone. The major velocity increment of 1000 m/s is measured at a depth of 3260 m at 100% Sw as shown in Fig. 26(a). There is no significant change in shear wave velocity because it is independent of fluid. Due to a change in water saturation, the density changes. Therefore, shear wave velocity behaves slightly differently at different saturation levels. The maximum increase of 30 m/s shear wave velocity is observed at a depth of 3254 m as shown in Fig. 26(b). The water is denser than gas therefore density is increased at both saturation level due to water substitution. The maximum difference of 0.09 g/cc density is observed at a depth of 3255 m as shown in Fig. 26(c).

**Fig. 27:**
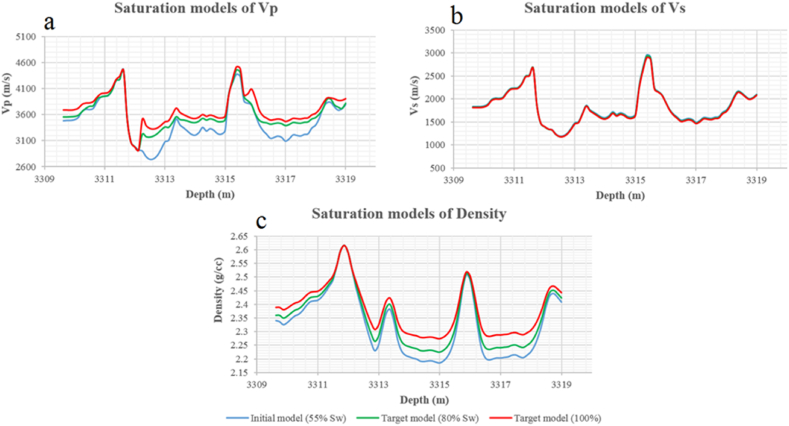
The variations of seismic velocities and density at 80% and 100% Sw level after Gassmann fluid substitution: (a) Primary wave velocity, (b) Secondary wave velocity, (c) Saturated density.

The second zone in Sawan-01 well is selected at the depth of 3309–3319 m. The inceptive saturation of water secured from the petrophysical analysis is 55%. The desired saturation levels are 80% and 100% water saturation in this zone. The P-wave velocity is increasing with an increase in water saturation level. The maximum velocity increment of 580 m/s is measured at a depth of 3212.4 m at 100% Sw shown in Fig. 27(a). The shear wave velocity is not much affected by fluid substitution. The small change in shear wave velocity is due to density. Due to water substitution, the density is increased. Therefore, shear wave velocity is slightly decreased. The maximum decrease of 50 m/s shear wave velocity is measured at a depth of 3315.4 m as shown in Fig. 27(b). The water density is greater as compared to the gas density therefore density is increased at both saturation level due to water substitution. The maximum difference of 0.0885 g/cc in density is examined at a depth of 3314.85 m as shown in Fig. 27(c).

**Fig. 28:**
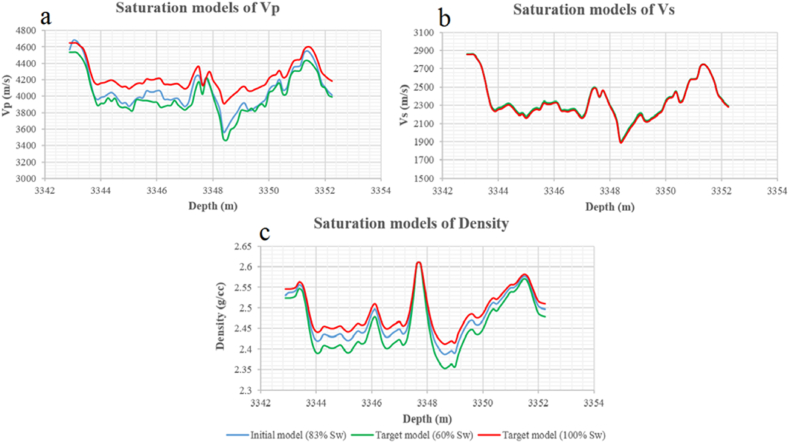
The variations of seismic velocities and density at 60% and 100% Sw level after Gassmann fluid substitution: (a) Primary wave velocity, (b) Secondary wave velocity, (c) Saturated density.

The third zone is marked at a depth of 3342.8–3352.3 m in Sawan-01 well. The measured water saturation at the initial level is 83%. The target saturation levels are 60% and 100% water saturation in this zone. The p-wave velocity is increased with an increase in the Sw from the initial level to 100%. The maximum velocity increment of 349 m/s is observed at a depth of 3348.4 m shown in Fig. 28(a). The shear wave velocity is slightly increased due to the change in density at different saturation levels. The maximum increase of 10 m/s shear wave velocity is observed at 3348 m depth as shown in Fig. 28(b). The density is also changing with a change in water saturation. The maximum difference of 0.0246 g/cc in density is observed at a depth of 3348.6 m as shown in Fig. 28(c).

### 4.8 Cross plots analysis

The Vp/Vs ratio can easily distinguish the fluid types in the reservoir zone. The acoustic impedance can be utilized to identify the type of lithology. However, the cross plot of the AI and Vp/Vs ratio can differentiate the fluid type and lithology more accurately than individual parameters. The cross plot of lambda-rho and Mu-rho can distinguish the fluid type and lithology [51].

**Fig. 29:**
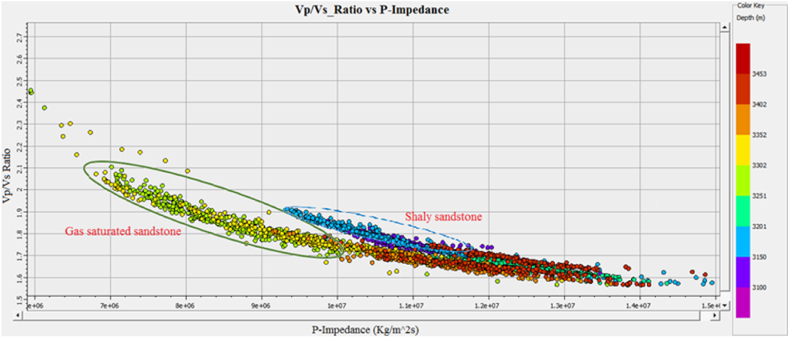
P-impedance vs. Vp/Vs ratio cross plot of Sawan-01 well.

Cross plots of Sawan-01 well: Fig. 29 demonstrates the cross plot of P-impedance and Vp/Vs ratio of Lower Goru Formation. The reservoir zones selected from the petrophysical analysis are at a depth of 3252–3319 m in Sawan-01 well. The cluster of green and yellow color data points indicates an increase in P-impedance. This increase in P-impedance indicates the compaction of lithology and a decrease in porosity. The increase in Vp/Vs ratio with low P-impedance indicates the presence of hydrocarbon [52]. The blue color points are falling in the shale area where Vp/Vs ratio and P-impedance values are high. The petrophysical analysis also shows a decrease in porosity with an increase in depth, as shown in Table 3.

**Fig. 30:**
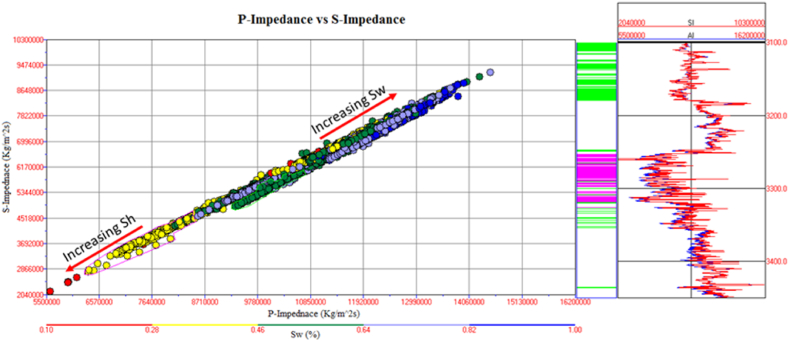
P-impedance vs. S-impedance cross plot of Sawan-01 well.

The cross plot of P-impedance and S-impedance is used to identify the fluids variation trends [52], as shown in Fig. 30. The yellow color data points with low water saturation, P-impedance, and S-impedance indicates gas saturated sandstone. The data points encircled by pink color indicate a gas reservoir at a depth of 3252–3319 m depth. In the petrophysical analysis, this zone is also marked as a reservoir zone. The data points with high water saturation, P-impedance, and S-impedance indicate the brine-saturated sandstone shown in Fig. 30.

**Fig. 31:**
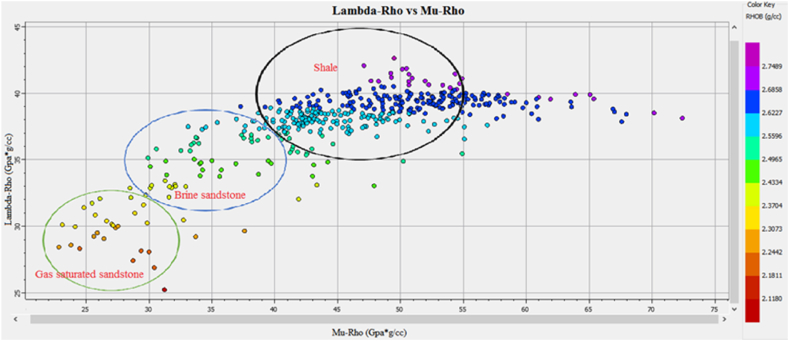
The LR versus MR cross plot of zone 1 and zone 2 in Sawan-01 differentiating fluids and lithology.

The cross plot of lambda-rho versus mu-rho of zone1 and zone 2 at a depth of 3252–3319 m in Sawan-01 well is shown in Fig. 31. The data points within the green circle are representing gas enriched sandstone where both Lambda-rho and Mu-rho are low. The data points encircled by a blue circle are indicating brine sandstone. The high Mu-rho and Lambda-rho data, encircled by black color are shales representing points [52]. In terms of significance, each of these parameters (AI-SI, AI - Vp/Vs, lanbda-rho vs. Mu-rho) provides unique information about the subsurface and can be used in different ways to interpret geophysical data. For example, AI and SI are often used in conjunction with seismic inversion techniques to create high-resolution images of the subsurface, while Vp/Vs is frequently used to identify hydrocarbon reservoirs and structural traps. Lambda-rho and mu-rho are often used in rock physics modeling to predict rock properties and aid in the interpretation of seismic data [13,41,53].

**Fig. 32:**
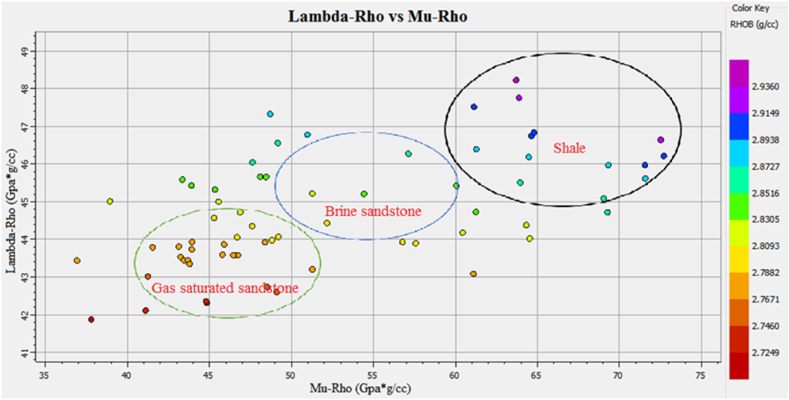
The LR versus MR cross plot of zone 3 in Sawan-01 differentiating fluids and lithology.

The cross plot of Lambda-rho versus Mu-rho of zone 3 at a depth of 3342–3352 m in Sawan-01 well is shown in Fig. 32. The low density and lambda-rho data points encircled by green color are indicating gas sandstone. The turquoise color points and data points encircled by blue color are indicating brine sandstone. The data points with high LMR values are shale representing data points.

**Fig. 33:**
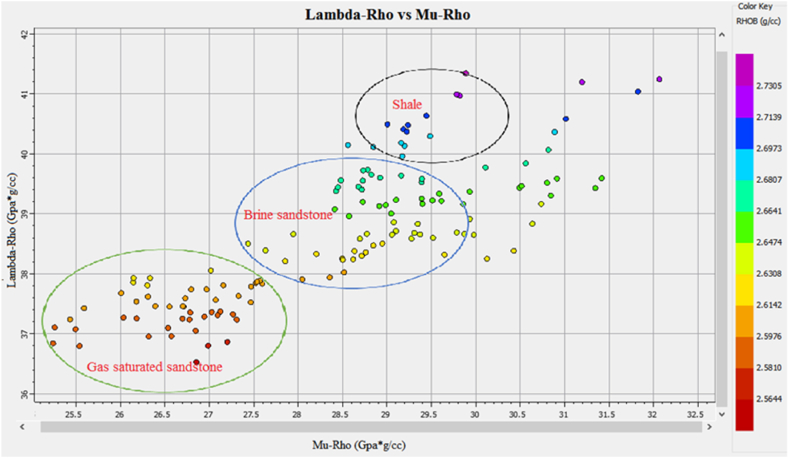
LR versus MR cross plot of zone 4 in Sawan-01 differentiating fluids and lithology.

The cross plot of Lambda-rho versus Mu-rho of zone 4 at a depth of 3369–3390 m in Sawan-01 well is shown in Fig. 33. The low density and low Lambda-rho data points encircled by green color are indicating gas sandstone. The data points encircled by blue color are indicating brine sandstone. The data points with high LMR values are shale representing points.’

**Fig. 34:**
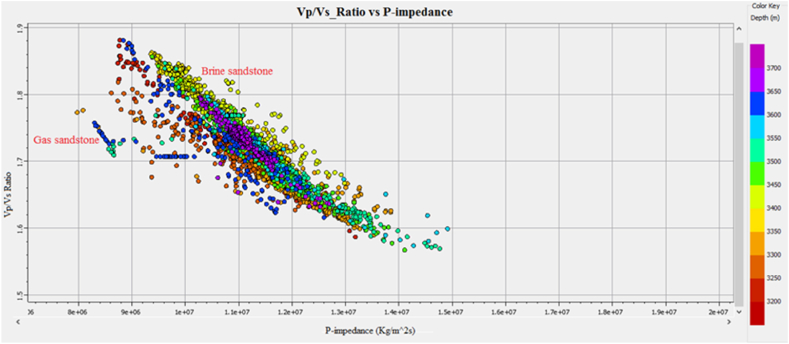
P-impedance vs. Vp/Vs ratio cross plot of Judge-01 well.

**Cross plots of Judge-01 well**: The petrophysical analysis of the reservoir zone in this well is selected at a depth of 3636–3652 m. The blue color data points in Fig. 34 showing low P-impedance and high Vp/Vs ratio are indicating hydrocarbon presence. The pale green and golden color data points showing a high Vp/Vs ratio and high P-impedance fall between brine sandstone and gas sandstone area [52]. The parrot green and turquoise color data points indicate the compacted lithology with low Vp/Vs ratio and high P-impedance. The porosity is decreasing on the P-impedance axis with increasing depth. The petrophysical analysis also indicates a decrease in porosity with an increase in depth in this zone, as shown in Table 4.

**Fig. 35:**
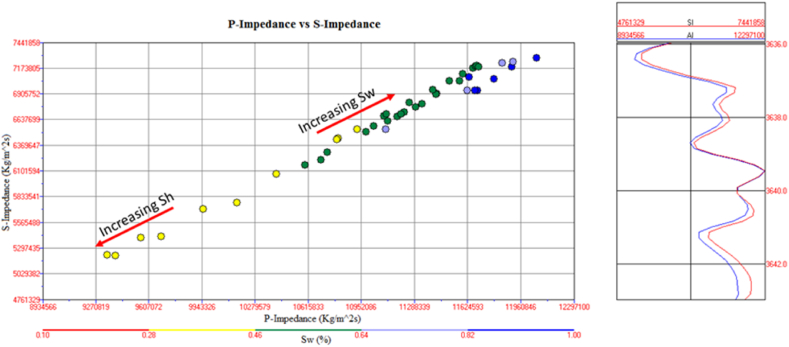
P-impedance vs S-impedance cross plot of Judge-01 well.

The cross plot of P-impedance (PI) and S-impedance (SI) of reservoir zone 1 in Judge-01 well is shown in Fig. 35. The yellow color data points with low saturation of water, P-impedance, and S-impedance indicate gas saturated sandstone. In the petrophysical analysis, this zone is also marked as a reservoir zone shown in Fig. 15. The data points with high water saturation, P-impedance and S-impedance are indicating the brine saturated sandstone as shown in Fig. 35.

**Fig. 36:**
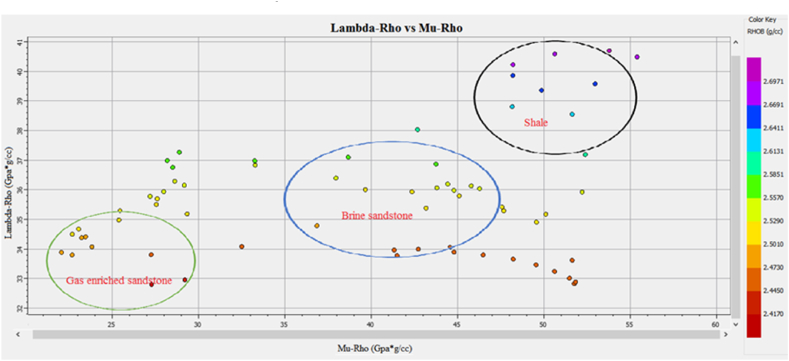
The LR versus MR cross plot of zone 1 in Judge-01 differentiating fluids and lithology.

Fig. 36 shows the cross plot of reservoir 1 at a depth of 3636–3643 m in Judge-01 well. The low-density data points encircled by green color indicates gas sandstone having low Lambda-rho and Mu-rho values. The data points with high Lambda-rho and Mu rho values encircled by blue color are indicating brine sandstone. The high values of Lambda-rho and Mu-rho encircled by black color are shale.

**Fig. 37:**
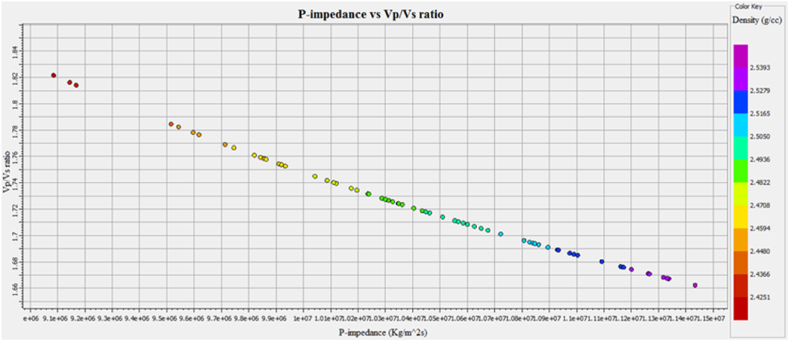
P-impedance vs. Vp/Vs ratio cross plot of C sand.

**Cross Plot of C sand horizon**: The cross plot of P-impedance versus seismic velocities ratio for C sand is shown in Fig. 37. The cross plot shows an inverse relation between impedance and seismic velocities ratio. The low Vp/Vs ratio and high P-impedance value indicate a decrease in porosity with an increase in density. This decreasing porosity trend indicates shaly sandstone for C sand.

**Fig. 38:**
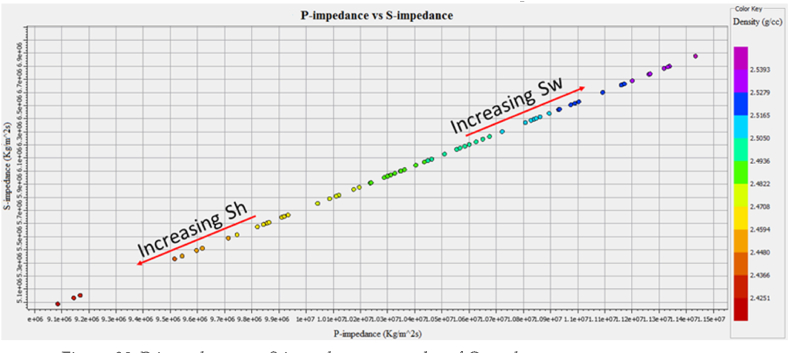
P-impedance vs. S-impedance cross plot of C sand.

The cross plot of PI versus SI for C sand is shown in Fig. 38. The data point with low values of P-impedance and S-impedance with low density are indicating gas saturated sandstone. The data points with high P-impedance and S-impedance with high-density values indicate brine saturated sandstone as shown in Fig. 38.

## 5 Conclusions

This integrated study of 2D-seismic and well logs data performed seismic interpretation, petrophysical analysis, lithology, and mineralogical identification, seismic attributes and rock physics conclude that.

• The seismic sections and contour maps show normal faulting having horst and graben structures. The normal faults are dipping in a NE-SW direction, and they are deep-rooted. These faults are also providing structural traps for hydrocarbon preservation.
• The petrophysical analysis shows significant hydrocarbon potential in Sawan-01 well with 61% hydrocarbon saturation in zone 1 and zone 2. Zone 1 at a depth of 3636–3643 m in Judge-01 also shows hydrocarbon potential with 48% hydrocarbon saturation. The presence of different clay types, i.e., glauconite, illite and biotite indicates a marine depositional environment and results in high GR values.
• The rock physics analysis shows the presence of gas-enriched sandstone in the area. The low P-impedance and Vp/Vs ratio ratios are indicating gas sandstone. The shaly sandstone is indicated by low Vp/Vs ratio and high P-impedance values. The cementation of the reservoir is increasing with the increase in depth resulting in low porosity values.

## Author contribution statement

Muhsan Ehsan, Muhammad Arslan Shakeel Toor, and Muhammad Iqbal Hajana: Conceived and designed the experiments; Performed the experiments; Analyzed and interpreted the data; Contributed reagents, materials, analysis tools or data; Wrote the paper. Nadhir Al-Ansari, Amjad Ali, and Ahmed Elbeltagi: Contributed reagents, materials, analysis tools or data; Wrote the paper.

## Funding statement

This research did not receive any specific grant from funding agencies in the public, commercial, or not-for-profit sectors.

## Data availability statement

The seismic and well data used to support the findings of this study are restricted by DGPC in order to protect data confidentiality. The datasets presented in this article are not readily available because the data used for this research is highly confidential and is the property of the DGPC. It can only be provided to university students for research purposes with permission from the DGPC.

## Declaration of interest's statement

The authors declare no conflict of interest.
